# Development and Validation of a Seven-Gene Signature for Predicting the Prognosis of Lung Adenocarcinoma

**DOI:** 10.1155/2020/1836542

**Published:** 2020-08-17

**Authors:** Yingqing Zhang, Xiaoping Zhang, Xiaodong Lv, Ming Zhang, Xixi Gao, Jialiang Liu, Yufen Xu, Zhixian Fang, Wenyu Chen

**Affiliations:** ^1^Department of Respiration, The First Hospital of Jiaxing, Affiliated Hospital of Jiaxing University, Jiaxing 314000, China; ^2^Department of Science and Education, The First Hospital of Jiaxing, Affiliated Hospital of Jiaxing University, Jiaxing 314000, China; ^3^Department of Oncology, The First Hospital of Jiaxing, Affiliated Hospital of Jiaxing University, Jiaxing 314000, China

## Abstract

**Background:**

Prognosis is a main factor affecting the survival of patients with lung adenocarcinoma (LUAD), yet no robust prognostic model of high effectiveness has been developed. This study is aimed at constructing a stable and practicable gene signature-based model via bioinformatics methods for predicting the prognosis of LUAD sufferers.

**Methods:**

The mRNA expression data were accessed from the TCGA-LUAD dataset, and paired clinical information was collected from the GDC website. R package “edgeR” was employed to select the differentially expressed genes (DEGs), which were then used for the construction of a gene signature-based model via univariate COX, Lasso, and multivariate COX regression analyses. Kaplan-Meier and ROC survival analyses were conducted to comprehensively evaluate the performance of the model in predicting LUAD prognosis, and an independent dataset GSE26939 was accessed for further validation.

**Results:**

Totally, 1,655 DEGs were obtained, and a 7-gene signature-based risk score was developed and formulated as risk_score = 0.000245∗NTSR1 + (7.13*E* − 05)∗RHOV + 0.000505∗KLK8 + (7.01*E* − 05)∗TNS4 + 0.000288∗C1QTNF6 + 0.00044∗IVL + 0.000161∗B4GALNT2. Kaplan-Meier survival curves revealed that the survival rate of patients in the high-risk group was lower in both the TCGA-LUAD dataset and GSE26939 relative to that of patients in the low-risk group. The relationship between the risk score and clinical characteristics was further investigated, finding that the model was effective in prognosis prediction in the patients with different age (age > 65, age < 65) and TNM stage (N0&N1, T1&T2, and tumor stage I/II). In sum, our study provides a robust predictive model for LUAD prognosis, which boosts the clinical research on LUAD and helps to explore the mechanism underlying the occurrence and progression of LUAD.

## 1. Introduction

Lung cancer is a kind of malignant tumor with the morbidity (13% both in male and female) and mortality (24% in male and 23% in female), respectively, ranking second and top worldwide, according to the latest data released in *A Cancer Journal for Clinicians* [1]. Lung cancer can be classified into small-cell lung cancer (SCLC) and non-small-cell lung cancer (NSCLC), of which NSCLC sufferers are in the majority of the total lung cancer cases (around 80%). Lung adenocarcinoma (LUAD), the main histological subtype of NSCLC, takes up over 40% among the overall lung cancer morbidity [2]. Given that around 80% of patients with lung cancer are diagnosed in middle and advanced stages, surgery is no more an available option, resulting in unfavorable outcomes with a 5-year overall survival (OS) rate of nearly 17% [3, 4]. While distant metastasis and relapse are main causes of poor cancer treatment and prognosis [5, 6], identification of cancer-associated genes and independent prognostic factors as well as investigation of their impact on tumor progression and prognosis is beneficial for the implementation of precision medicine and helps to raise the cure rate and improve the prognosis. With the development of gene chip technology and RNA sequencing, gene expression profiles have been widely applied in the prediction of LUAD prognosis. For example, PHLPP2 has been reported as a novel biomarker in NSCLC metastasis and prognosis [7]. Thyroid transcription factor-1 is considered as a prognostic marker indicating the presence or absence of EGFR-sensitizing mutations in stage IV LUAD [8]. And the elevated CX3CL1 mRNA expression is found to be a positive factor involved in LUAD prognosis [9]. However, due to the variety of methods, experimental platforms, batch effects, or other factors, discrepancy appears in the genes screened for prognosis prediction. Besides, the prognostic models constructed might be only practicable in the current experimental samples, while the performance in other independent datasets is less pronounced. Therefore, it is urgent to find a model that is practicable in various datasets, making its value realized in different clinical researches.

In the present study, HTSeq-Counts data of LUAD comprising 522 tumor samples and 58 normal samples were accessed from the TCGA database. Based on the data, survival-associated genes were selected using univariate COX regression analysis, after which the Lasso regression model was constructed to rule out the genes of a relatively stronger correlation to prevent model overfitting. Afterwards, a series of multivariate COX regression models were established, and the optimal model was identified in line with the Akaike Information Criterion (AIC). To validate and evaluate the performance of the model in predicting LUAD prognosis, various aspects were taken into account, finding that the model was effective in the training set and testing set, and its performance in patients with different age and TNM stage was validated to be good as well. Furthermore, the model also exhibited a good ability in predicting the prognosis of LUAD patients in an independent dataset GSE26939. To sum, our study constructs a robust gene signature-based model available for predicting the prognosis of LUAD patients, which helps the clinical research on LUAD and lays a foundation for the future investigation on the molecular mechanism underlying LUAD occurrence and progression.

## 2. Methods and Materials

### 2.1. Data Collection and Preprocessing

HTSeq-Counts data of LUAD (including 522 tumor samples and 58 normal samples) were obtained from the TCGA database (https://portal.gdc.cancer.gov/) and then used for differential analysis with the aid of R package “edgeR” (∣logFC | ≥2, adj. FDR < 0.05). The corresponding clinical information of TCGA-LUAD patients was collected in the GDC website (https://portal.gdc.cancer.gov/). Patients who were followed up less than 30 days were excluded in this study, and totally, 460 TCGA-LUAD patients were included eventually. Besides, to further verify the validity of the prognostic model, an independent dataset GSE26939 (including 115 patients with LUAD) and matched clinical information were accessed from the GEO database (https://www.ncbi.nlm.nih.gov/geo/).

### 2.2. Candidate Gene Selection

Differentially expressed genes (DEGs) screened out by “edgeR” were randomized into the training set and testing set (5 : 5) and then subjected to univariate COX regression analysis for identifying the genes associated with the survival of patients with LUAD. The Lasso regression model was employed to further analyze these survival-related genes to exclude the genes with a relatively higher correlation, contributing to the decrease in the complexity of the prognostic model [10] and helping to find the optimal signature genes.

### 2.3. Prognostic Model Construction

Candidate genes selected by Lasso regression analysis were used to construct multivariate COX models, and the Akaike Information Criterion (AIC) was referenced to find the optimal prognostic model.

### 2.4. Stability and Validity Verification

Patients in the training set and testing set were conferred a risk score and grouped into the high-risk group and the low-risk group based on the median score. The Kaplan-Meier method was conducted to compare the survival of patients in two groups, and log-rank was performed to calculate the *p* value. Meanwhile, ROC analysis was carried out to analyze the performance of the model in predicting the prognosis of LUAD patients, and an independent dataset GSE26939 was applied for the verification of the model's validity.

## 3. Results

### 3.1. Identification of Candidate Genes

**Figure 1:**
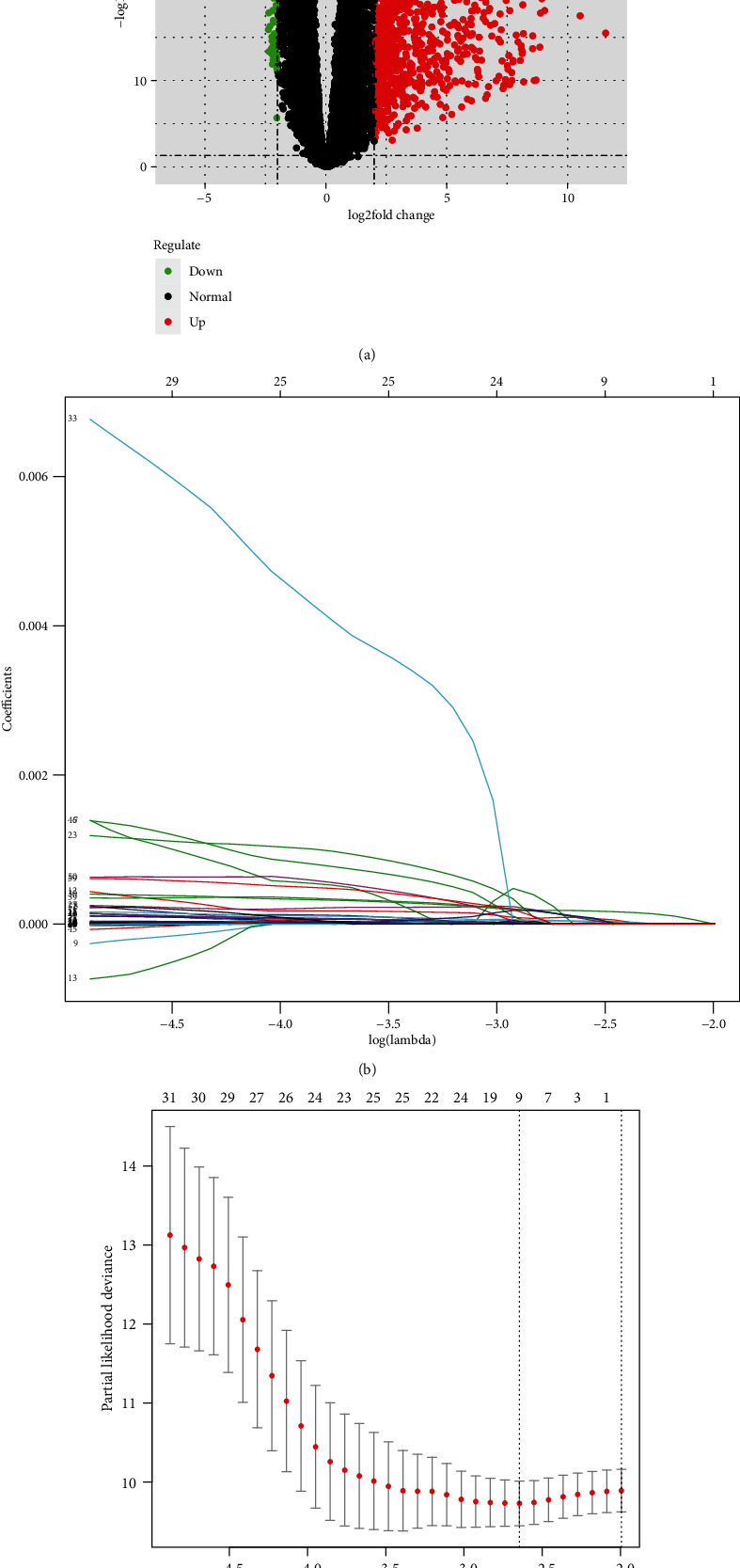
Candidate gene selection. (a) Volcano plot of the DEGs in the TCGA-LUAD dataset; (b) regression coefficients in Lasso regression analysis; (c) selection of lambda in the Lasso regression model through 10-fold crossvalidation method.

In total, 1,655 DEGs were obtained via differential analysis based on the TCGA-LUAD dataset (Figure 1(a)) and randomly assigned to the training set and testing set (5 : 5). Univariate COX analysis was performed to screen survival-related genes from the training set with the cut-off set as *p* value = 0.01, and initially, 60 genes were screened out as shown in Supplementary Table 1 (the top 20 genes associated with survival are listed in Table 1). Subsequently, these genes were analyzed in a Lasso regression model. Genes with a relatively higher correlation were removed to lower the complexity of the prognostic model, and finally, 9 candidate signature genes were identified, namely, NTSR1, RHOV, KLK8, TNS4, C1QTNF6, FAM83A, IVL, B4GALNT2, and CREG2 (Figures 1(b) and 1(c)).

### 3.2. Construction of a 7-Gene Signature-Based Prognostic Model for LUAD

A series of multivariate COX models were constructed based on the candidate genes, and the optimal model was then selected in line with AIC as shown in Table 2. A 7-gene signature-based risk score formula was established as Risk score = 0.000245∗NTSR1 + (7.13*E* − 05)∗RHOV + 0.000505∗KLK8 + (7.01*E* − 05)∗TNS4 + 0.000288∗C1QTNF6 + 0.00044∗IVL + 0.000161∗B4GALNT2.

### 3.3. Evaluation of the 7-Gene Signature-Based Model in Predicting the Survival of LUAD Patients

**Figure 2:**
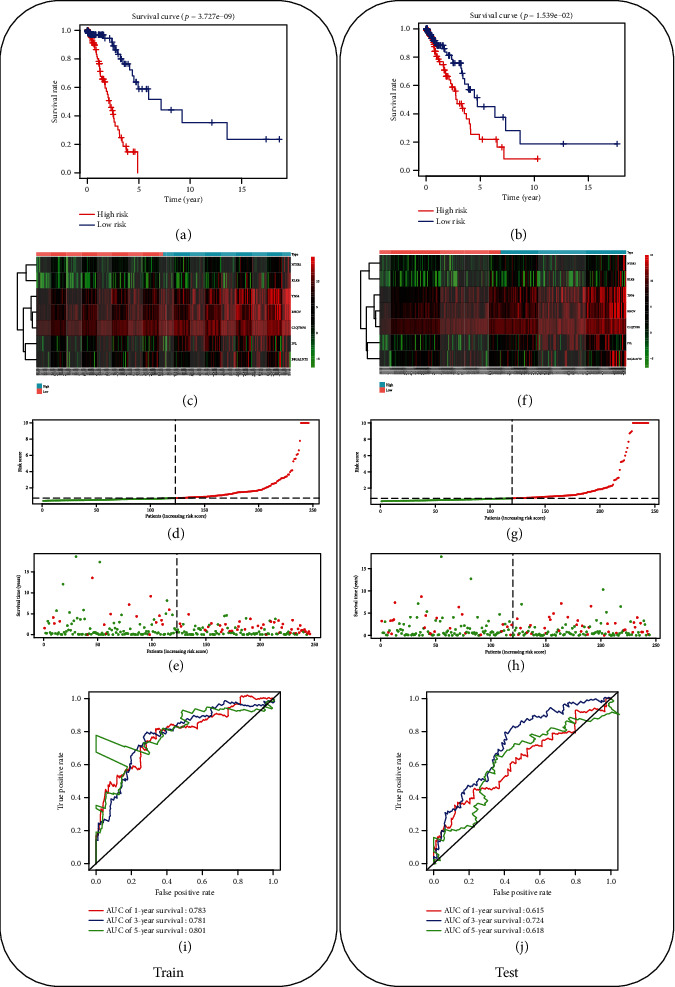
Evaluation of the 7-gene signature-based model in predicting the prognosis of LUAD patients. (a, b) The survival time between the patients of the high- and low-risk groups within the training set and testing set was analyzed by Kaplan-Meier analysis; (c–e) heat map for the 7 genes, as well as the risk score distribution and survival of patients in the training set; (f, g, h) heat map for the 7 genes, as well as the risk score distribution and survival of patients in the testing set; (i, j) ROC curves of the 7-gene signature-based model in two sets.

Based on the formula, the 7-gene signature-based risk score of each patient in the training set and testing set was calculated, and patients were classified into the high-risk group and the low-risk group according to the median score. Kaplan-Meier curves and log-rank test were used to compare the survival of the two groups in two independent sets, finding that patients in the high-risk group had poorer survival relative to those in the low-risk group in both sets (*p* < 0.05) (Figures 2(a) and 2(b)). To better know the expression level of the 7 genes, risk score distribution, and survival of the patients in two sets, data in the training set and testing set were obtained and plotted in Figures 2(c)–2(e) and 2(f)–2(h), respectively.

ROC analysis was conducted using the survivalROC package for the verification of the model performance in the training set and testing set. AUC values of 1-, 3-, and 5-year survival were calculated, with those in the training set as 0.783, 0.781, and 0.801 (Figure 2(i)) and in the testing set as 0.615, 0.724, and 0.618 (Figure 2(j)), respectively. Taken together, the 7-gene signature-based model was demonstrated to be capable of predicting the prognosis of LUAD patients.

### 3.4. Verification of Stability and Validity of the Prognostic Model for LUAD with an Independent Dataset GSE26939

**Figure 3:**
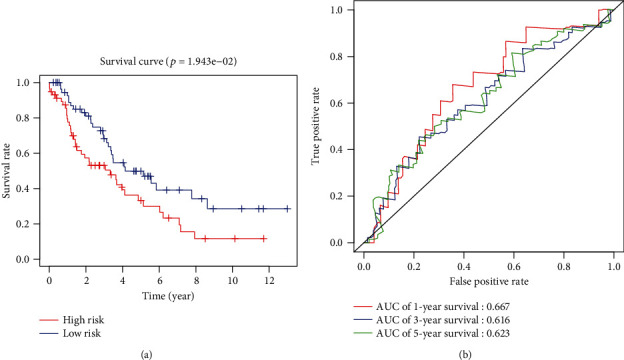
Verification of stability and validity of the prognostic model for LUAD with an independent dataset GSE26939. (a) Survival difference between the patients in the high- and low-risk groups was revealed by Kaplan-Meier analysis; (b) ROC curves of the 7-gene model using the independent dataset.

An independent dataset GSE26939 from the GEO database was applied to further verify the validity and stability of the 7-gene model. The same as the above procedures, patients were divided into the high-risk and low-risk groups based on the median risk score, and survival comparison was performed using Kaplan-Meier as shown in Figure 3(a), indicating the lower survival rate in the patients of the high-risk group (*p* < 0.05). Thereafter, ROC analysis was performed for further verification, with the AUC values of 1-, 3-, and 5-year survival of 0.667, 0.616, and 0.623 (Figure 3(b)), respectively. Collectively, this 7-gene model was practicable in other independent datasets.

### 3.5. Prognostic Impact of the Model on Clinical Characteristics

**Figure 4:**
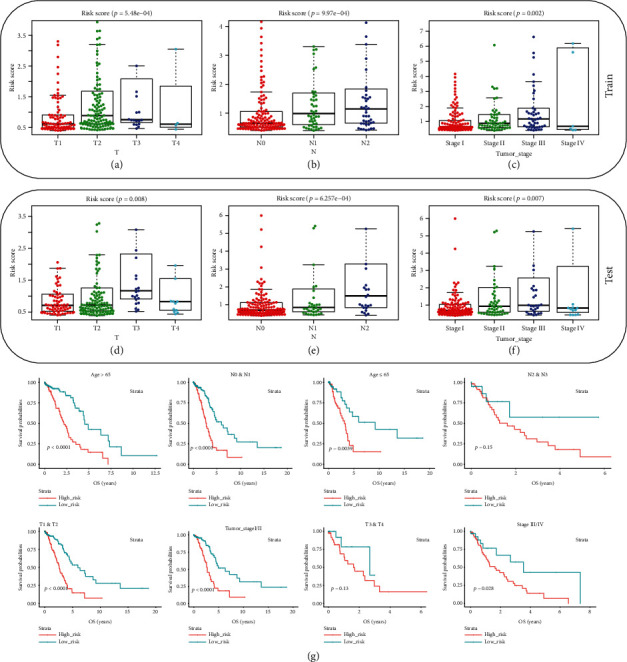
Prognostic impact of the model in clinical characteristics. The correlation of the 7-gene signature-based risk score with the TNM stage was analyzed in the (a–c) training set and (d–f) testing set. (g) OS curves were plotted for validation of the predictive ability of the 7-gene signature-based risk score in patients with different age and TNM stage.

To further discuss the correlation of the 7-gene signature-based risk score with the TNM (Tumor Node Metastasis) stage and overall survival (OS) of LUAD patients, matched clinical information of the training set and testing set was collected and is listed in Tables 3 and 4. The relationship between the risk score and TNM stage was explored, revealing that the risk score was significantly associated with pathologic T, N, and tumor stages of patients in both the training set (Figures 4(a)–4(c)) and testing set (Figures 4(d)–4(f)) (*p* < 0.05). Moreover, the performance of the model in predicting the prognosis of patients with different clinical characteristics in the two sets was investigated (Figure 4(g)), finding good performance on patients in different age and clinical stage (age > 65, age < 65, N0&N1, T1&T2, and tumor stage I/II). While in the independent dataset GSE26939, such correlation was less pronounced (Supplementary Table 2). Altogether, this 7-gene signature-based risk score model was a useful prognosis predictor in patients with different clinical characteristics and could be served as a novel biomarker in LUAD treatment.

## 4. Discussion

Lung cancer, with its mortality ranking top globally, often appears to be in middle and advanced stages when being initially diagnosed in most patients, and surgery is no more useful. In addition, the treatment and prognosis of patients are mainly affected by distant metastasis and relapse. Thus, it is highly important to build a predictive model characterized by high stability and validity for better early diagnosis, medication guidance, and prognosis prediction. At present, many studies have focused on the construction of prognostic models for LUAD treatment. For instance, Li et al. suggested that clinical immune characteristics were a promising biomarker that could be used to evaluate OS of nonsquamous NSCLC patients (including early disease) [11]. Park et al. tried to construct a gene signature-based prognostic model for LUAD [12], and in 2016, Shukla et al. proposed the first RNA-seq-based prognostic signature through analyzing the RNA-seq and clinical data, making an attempt to develop a potent predictive tool for LUAD prognosis [13]. Despite the extensive research on signature genes used for LUAD prognosis, models with robust prediction capability have yet to be successfully constructed. Besides, with the development of high-throughput sequencing, more gene datasets of LUAD should be employed into new studies.

In our study, seven LUAD survival-related genes were identified, including NTSR1, RHOV, KLK8, TNS4, C1QTNF6, IVL, and B4GALNT2. These 7 signature genes were obtained from the HTSeq-Counts in the TCGA-LUAD dataset using univariate COX, Lasso regression, and multivariate COX analyses. Sequentially, the risk score based on the 7-gene signature was established and formulated as risk_score = 0.000245∗NTSR1 + (7.13*E* − 05)∗RHOV + 0.000505∗KLK8 + (7.01*E* − 05)∗TNS4 + 0.000288∗C1QTNF6 + 0.00044∗IVL + 0.000161∗B4GALNT2. As reported, most of these 7 genes are closely related to cancer progression. For example, NTSR1 (Neurotensin Receptor 1) has been reported as a potential prognostic biomarker for surgically resected stage I LUAD [14] and prostate cancer [15]. RHOV (Ras Homolog Family Member V) has been verified to be highly expressed in NSCLC and can serve as a signature gene in LUAD prognosis [16]. KLK8 (Kallikrein Related Peptidase 8) has presented its research value in the prognosis of various cancers, such as lung cancer [17], ovarian cancer [18], breast cancer [19], colon cancer, and rectal cancer [20]. Moreover, TNS4 (Tensin 4) has been found to be upregulated in LUAD and able to predict poor prognosis, and it has been observed to be mediated by miR-150-3p [21]. Meanwhile, another study indicated that the aberrant methylation of TNS4 is significantly associated with the OS of LUAD patients [22]. C1QTNF6 (C1q/tumor necrosis factor-related protein 6), a member of the CTRP family, has shown its potential as an independent predictor for the prognosis of LUAD sufferers [23]. Additionally, although the role of B4GALNT2 (Beta-1,4-N-Acetyl-Galactosaminyltransferase 2) in LUAD has not been investigated, it has been observed to be highly related to gastric cancer metastasis [24]. However, the association between IVL (Involucrin) and the progression of LUAD has not been reported, which requires further study in the future. In view of the above studies, we could conclude that some of these signature genes exhibit a certain relationship with the prognosis of other cancers.

During the research, each patient in the training set and testing set was conferred a risk score and classified into the high-risk group and the low-risk group according to the median score. As suggested in OS curves, patients in the high-risk group had poorer survival. ROC curves were plotted, and the AUC values of 1-, 3-, and 5-year survival in two sets were all above 0.6, indicating that the 7-gene signature-based risk score model was capable of predicting LUAD prognosis. Notably, similar results were found in an independent dataset GSE26939 from the GEO database, demonstrating the validity and practicality of this 7-gene model. Furthermore, the association between this model and clinical characteristics of LUAD patients was explored, finding that the model functioned well in predicting the prognosis of patients with different age (age > 65, age < 65) and TNM stage (N0&N1, T1&T2, and tumor stage I/II), while the effect in the GSE26939 was less remarkable.

## 5. Conclusions

In conclusion, we obtained 1,655 DEGs from the TCGA-LUAD dataset using the “edgeR” package and constructed a prognostic 7-gene signature-based model (containing NTSR1, RHOV, KLK8, TNS4, C1QTNF6, IVL, and B4GALNT2, seven genes) through univariate COX, Lasso, and multivariate COX regression analyses. The robust model we built helps to advance the clinical research on LUAD and better understand the mechanism underlying LUAD occurrence and progression.

## Figures and Tables

**Table 1:** Top 20 genes associated with survival.

ID	HR	HR.95L	HR.95H	*p* value
FAM83A	1.00004	1.000026	1.000053	8.36*E* − 09
RHOV	1.000144	1.000093	1.000196	4.16*E* − 08
TNS4	1.000136	1.000083	1.00019	5.85*E* − 07
C1QTNF6	1.000485	1.000288	1.000683	1.51*E* − 06
COL7A1	1.000145	1.000079	1.000211	1.86*E* − 05
TRPA1	1.002849	1.001522	1.004177	2.53*E* − 05
CREG2	1.000977	1.000508	1.001445	4.34*E* − 05
UNC5D	1.000503	1.000261	1.000746	4.69*E* − 05
INHA	1.000112	1.000056	1.000167	8.79*E* − 05
AHNAK2	1.000072	1.000035	1.000108	0.000107
OLFM4	1.000025	1.000012	1.000037	0.00011
KLK8	1.000654	1.000312	1.000996	0.000177
FAM83B	1.001885	1.000896	1.002875	0.000186
KRT6A	1.000015	1.000007	1.000022	0.000285
MUCL1	1.000166	1.000076	1.000255	0.000305
MFI2	1.000104	1.000047	1.000162	0.000381
ERO1L	1.000042	1.000019	1.000065	0.000388
PSG5	1.053914	1.023725	1.084994	0.000398
NTSR1	1.000394	1.000171	1.000616	0.00054
TMPRSS11E	1.000327	1.000141	1.000513	0.000562

**Table 2:** The 7 genes in the optimal multivariate COX regression model.

ID	Coefficient	HR	HR.95L	HR.95H	*p* value
NTSR1	0.000244865	1.000244895	0.999995	1.000495	0.05509
RHOV	7.13*E* − 05	1.000071315	0.999989	1.000153	0.08795
KLK8	0.000505343	1.000505471	1.000028	1.000983	0.037875
TNS4	7.01*E* − 05	1.000070108	0.999986	1.000154	0.103366
C1QTNF6	0.000287673	1.000287714	1.000022	1.000553	0.033585
IVL	0.000440486	1.000440583	1.000203	1.000678	0.000272
B4GALNT2	0.000161186	1.000161199	1.000032	1.00029	0.014511

**Table 3:** Clinical information of LUAD patients in the training set.

	Low risk (*n* = 116)	High risk (*n* = 117)	*p* value
*Age*			
<65	51 (44.0%)	59 (50.4%)	0.392
>65	65 (56.0%)	58 (49.6%)	
*Event*			
Yes	17 (14.7%)	40 (34.2%)	<0.001
No	99 (85.3%)	77 (65.8%)	
*Gender*			
Female	61 (52.6%)	65 (55.6%)	0.746
Male	55 (47.4%)	52 (44.4%)	
*T*			
T1	53 (45.7%)	33 (28.2%)	0.0246
T2	52 (44.8%)	71 (60.7%)	
T3	8 (6.9%)	12 (10.3%)	
T4	3 (2.6%)	1 (0.9%)	
*N*			
N0	85 (73.3%)	56 (47.9%)	<0.001
N1	16 (13.8%)	32 (27.4%)	
N2	15 (12.9%)	29 (24.8%)	
*Tumor stage*			
Stage I	75 (64.7%)	48 (41.0%)	0.0022
Stage II	22 (19.0%)	35 (29.9%)	
Stage III	15 (12.9%)	31 (26.5%)	
Stage IV	4 (3.4%)	3 (2.6%)	
*Smoking*			
≤40 pack years of smoke	71 (61.2%)	75 (64.1%)	0.748
>40 pack years of smoke	45 (38.8%)	42 (35.9%)	

**Table 4:** Clinical information of LUAD patients in the testing set.

	Low risk (*n* = 112)	High risk (*n* = 115)	*p* value
*Age*			
<65	41 (36.6%)	53 (46.1%)	0.189
>65	71 (63.4%)	62 (53.9%)	
*Event*			
Yes	22 (19.6%)	34 (29.6%)	0.114
No	90 (80.4%)	81 (70.4%)	
*Gender*			
Female	62 (55.4%)	59 (51.3%)	0.632
Male	50 (44.6%)	56 (48.7%)	
*T*			
T1	42 (37.5%)	30 (26.1%)	0.00148
T2	62 (55.4%)	57 (49.6%)	
T3	3 (2.7%)	20 (17.4%)	
T4	5 (4.5%)	8 (7.0%)	
*N*			
N0	94 (83.9%)	71 (61.7%)	<0.001
N1	13 (11.6%)	23 (20.0%)	
N2	5 (4.5%)	21 (18.3%)	
*Tumor stage*			
Stage I	77 (68.8%)	51 (44.3%)	0.00205
Stage II	20 (17.9%)	32 (27.8%)	
Stage III	9 (8.0%)	23 (20.0%)	
Stage IV	6 (5.4%)	9 (7.8%)	
*Smoking*			
≤40 pack years of smoke	75 (67.0%)	61 (53.0%)	0.045
>40 pack years of smoke	37 (33.0%)	54 (47.0%)	

## Data Availability

All the data in my manuscript is available.

## Supplementary Materials

Supplementary Materials Supplementary Table 1: survival-related genes. Supplementary Table 2: matched clinical info of patients in independent dataset GSE26939.
