# Supplementary material for: Development and Validation of a Seven-Gene Signature for Predicting the Prognosis of Lung Adenocarcinoma
Source: Biomed Res Int. 2020 Aug 17;2020:1836542. doi: 10.1155/2020/1836542 (PMC7641279; doi:10.1155/2020/1836542)
Supplement: Supplementary Materials — Supplementary Table 1: survival-related genes. Supplementary Table 2: matched clinical info of patients in independent dataset GSE26939. [file 1836542.f1.pdf]

Supplementary Table 1: Survival-related genes

ID	HR	HR.95L	HR.95H	P-value
FAM83A	1.00004	1.000026	1.000053	8.36E-09
RHOV	1.000144	1.000093	1.000196	4.16E-08
TNS4	1.000136	1.000083	1.00019	5.85E-07
C1QTNF6	1.000485	1.000288	1.000683	1.51E-06
COL7A1	1.000145	1.000079	1.000211	1.86E-05
TRPA1	1.002849	1.001522	1.004177	2.53E-05
CREG2	1.000977	1.000508	1.001445	4.34E-05
UNC5D	1.000503	1.000261	1.000746	4.69E-05
INHA	1.000112	1.000056	1.000167	8.79E-05
AHNAK2	1.000072	1.000035	1.000108	0.000107
OLFM4	1.000025	1.000012	1.000037	0.00011
KLK8	1.000654	1.000312	1.000996	0.000177
FAM83B	1.001885	1.000896	1.002875	0.000186
KRT6A	1.000015	1.000007	1.000022	0.000285
MUCL1	1.000166	1.000076	1.000255	0.000305
MFI2	1.000104	1.000047	1.000162	0.000381
ERO1L	1.000042	1.000019	1.000065	0.000388
PSG5	1.053914	1.023725	1.084994	0.000398
NTSR1	1.000394	1.000171	1.000616	0.00054
TMPRSS11E	1.000327	1.000141	1.000513	0.000562
ARHGAP11A	1.00049	1.00021	1.00077	0.000605
ARNTL2	1.000142	1.000059	1.000226	0.000823
KLF4	1.000195	1.00008	1.00031	0.000908
CYP27C1	1.001453	1.000594	1.002313	0.000908
CASP14	1.000805	1.000327	1.001283	0.000965
GJB3	1.000283	1.000114	1.000451	0.000992
BHMT	1.00464	1.001799	1.007489	0.001354
UGT2B11	1.000636	1.000247	1.001026	0.00137
SLC2A1	1.000035	1.000014	1.000057	0.001437
HAL	1.000287	1.00011	1.000463	0.001459
CYP24A1	1.000025	1.00001	1.000041	0.001642
DEPDC7	1.000705	1.000261	1.001149	0.001867
TINAG	1.001562	1.000574	1.002551	0.001931
SERPINA5	1.000644	1.000235	1.001054	0.002029
ASPM	1.000445	1.000162	1.000728	0.002043
GCLC	1.000025	1.000009	1.000042	0.002228
KLK6	1.000201	1.000072	1.000331	0.002282
RRM2	1.000162	1.000058	1.000266	0.002342

ACY3	1.000793	1.000282	1.001305	0.002375
IVL	1.000349	1.000122	1.000576	0.002619
CENPF	1.000186	1.000063	1.000309	0.002992
SLC6A15	1.000666	1.000225	1.001107	0.003075
MKI67	1.000135	1.000045	1.000225	0.003241
STRIP2	1.000304	1.000098	1.000509	0.00373
PPP2R2C	1.000266	1.000086	1.000446	0.003803
FRMD3	1.002736	1.000871	1.004604	0.004012
TNFSF11	1.001062	1.000338	1.001786	0.004026
GJB2	1.000105	1.000033	1.000177	0.004101
UPK1B	1.000082	1.000026	1.000138	0.004183
CPS1	1.00001	1.000003	1.000017	0.004312
SERPINB3	1.000203	1.000061	1.000345	0.005132
KRT16	1.000068	1.000019	1.000118	0.00661
B4GALNT2	1.000152	1.000041	1.000263	0.007181
DRP2	1.001752	1.000461	1.003044	0.007821
HMMR	1.000426	1.000109	1.000742	0.008357
IGF2BP1	1.000238	1.00006	1.000417	0.008725
KRT17	1.00002	1.000005	1.000036	0.00893
RGS20	1.00124	1.00031	1.002171	0.008956
SERPINB4	1.000881	1.000213	1.00155	0.009781

Supplementary Table 2: Matched clinical info of patients in independent dataset GSE26939

	Low risk(n=58)	High risk(n=57)	P-value
**age**			
<65	30 (51.7%)	23 (40.4%)	0.3
>=65	28 (48.3%)	34 (59.6%)	
**Event**			
Yes	29 (50.0%)	37 (64.9%)	0.153
No	29 (50.0%)	20 (35.1%)	
**gender**			
F	32 (55.2%)	30 (52.6%)	0.931
M	26 (44.8%)	27 (47.4%)	
**Stage**			
stage III	10 (17.2%)	9 (15.8%)	0.583
NA	5 (8.6%)	8 (14.0%)	
stage I	32 (55.2%)	30 (52.6%)	
stage II	9 (15.5%)	10 (17.5%)	
stage IV	2 (3.4%)	0 (0%)	
**grade**			

grade 1	4 (6.9%)	5 (8.8%)	0.308
grade2	33 (56.9%)	25 (43.9%)	
grade3	18 (31.0%)	25 (43.9%)	
Missing	3 (5.2%)	2 (3.5%)	
**smoking pack years**			
<=40 Pack Years of	26 (44.8%)	24 (42.1%)	0.834
>40 Pack Years of smoke	20 (34.5%)	22 (38.6%)	
Missing	12 (20.7%)	11 (19.3%)	
**EGFR**			
wild	32 (55.2%)	38 (66.7%)	0.18
mutant	8 (13.8%)	3 (5.3%)	
Missing	18 (31.0%)	16 (28.1%)	
**KRAS**			
wild	48 (82.8%)	40 (70.2%)	0.354
mutant	8 (13.8%)	12 (21.1%)	
Missing	2 (3.4%)	5 (8.8%)	
**tp53**			
wild	41 (70.7%)	34 (59.6%)	0.328
mutant	13 (22.4%)	18 (31.6%)	
Missing	4 (6.9%)	5 (8.8%)	
